# Screen-based behaviour in children is more than meets the eye

**DOI:** 10.4102/safp.v64i1.5374

**Published:** 2022-02-10

**Authors:** Alvin J. Munsamy, Verusia Chetty, Suvira Ramlall

**Affiliations:** 1 Discipline of Optometry, School of Health Sciences, College of Health Sciences, University of KwaZulu-Natal, Durban, South Africa; 2 Department of Physiotherapy, College of Health Sciences, University of KwaZulu-Natal, Durban, South Africa; 3 Department of Psychiatry, College of Health Sciences, University of KwaZulu-Natal, Durban, South Africa

**Keywords:** sedentary screen-based behavior, screen time, children, vision, physical health, mental well-being

## Abstract

Increased screen time (ST) in children is quickly becoming a public health concern as children are now reliant on technology for social interaction and educational development. The eye-health community has paid considerable attention to this in the recent literature, documenting it as digital eye strain. Continual close eye work and a lack of outdoor play contribute to digital eye strain and today’s myopia epidemic. This is a cause for concern for public health stakeholders insofar as it leads to sedentary, screen-based behaviour (SSB) in children. This results in a lack of physical activity and impacts both their bodies and their mental health. The potentially harmful effects of prolonged screen exposure on developing brains and bodies are likely to be unique and significant as physiological growth changes intersect with exponentially expanding e-platforms. While embracing the benefits of a highly digitalised world, we need to simultaneously mitigate the potential risks they pose to the health of growing children.

## Introduction

The reliance on devices, and the resulting screen time (ST), before and after the 2020 pandemic, is part of the ‘new normal’. This has resulted in children exhibiting various developmental conditions. The physiological effects of the use of devices appear to impact more than just children’s eyes although it is through the eye that devices are primarily accessed and which we associate with ST.^1,2^ It is time to recognise that the effects of sedentary, screen-based (SSB) behaviour extend beyond the eye health of children.^2^ The more time is spent on SSB, the greater the consequences for systemic health and fitness, with an increased incidence of obesity and cardiovascular disease in children.^2^ Historically, these were not associated with children before the 21st century. Screens also emit blue light, which affects sleep and causes inattention in children, further raising mental health concerns.^3,4^ The question arises whether parents and teachers are aware of this when allowing children to interact with their devices.

## Screen time and the vision of children

Screen time equates to near work and most often requires reading. Continual close eye work and a lack of outdoor play contribute to digital eye strain and today’s myopia epidemic.^5^ The issue with screens, compared to traditional books, is that screens are starting to reduce in size, as demonstrated by mobile phones and tablet devices.^3,6^ As a result, the working distance reduces to ensure clarity. The visual system then has to increase its accommodative efforts that, if this is continuous, lead to eyestrain.^3,6^ Screens can also cause eyestrain because of the glare emitted from the backlighting, which is not present with printed reading material.^6^ This is all exacerbated by prolonged ST, which is becoming problematic, especially for children’s developing eyes.

Although scientists have not shown a direct link between device use and the progression of myopia, indirect evidence points to some causal relationship. In 2020, Wang et al.^7^ found an increase in the prevalence of myopia in children, compared to prior years. They concluded that environmental factors, such as home confinement in 2020, were contributing factors.^8^ Screen time per se was not explicitly highlighted although it was a significant part of home confinement activities and during online schooling. This raises concerns regarding the effects of home confinement, which may lead to ‘quarantine myopia’, because of increased time spent reading indoors and less time spent outdoors^8^ during the COVID-19 pandemic.^7^ Compulsory breaks from ST need to be consciously implemented at home and at school to maintain proper development of the eyes.

Children from the predigital age were, historically, not of concern in terms of refractive errors^5^, and this may be attributed to a lifestyle without devices. Even just a few years ago, bigger, bulky, less mobile, less available desktops were set at a further working distance, compared to today’s devices. Learning, and even playing, in the digital age demands more indoor time and more ST, and this has led to an increase in unnatural refractive errors in this age group. When compounded with the genetics of parental myopia^5^, this generation is left most vulnerable.

Public health concern with eye health in children needs to be recognised by the mainstream healthcare fraternity. It requires a multidisciplinary approach, and we should not restrict our intervention to ST and eye health alone, as ST affects more than just the eye.

## Screen time and the physical health of children

Another increasing concern for public health stakeholders is that the increased ST causes sedentary behaviour in children and results in a lack of physical activity.^9^ ‘Sedentary behaviour is any waking behaviour characterised by an energy expenditure of ≤ 1.5 metabolic equivalents (METs), while in a sitting, reclining, or lying posture’.^10^ Often sedentary behaviour is associated with the use of electronic devices, such as smartphones/tablets, televisions and computers.^10^ This lack of physical activity, which challenges the health and well-being of children, had already been a national concern prior to the COVID-19 pandemic. Unfortunately, the pandemic saw children socially isolated and lockdown in many countries, leading to long periods confined indoors. Children sought online platforms to connect socially to maintain academic development and for entertainment. This meant increased ST and less physical activity.^11^ A growing concern with excessive ST in children is that it encourages sedentary behaviour and poor diet, which can inadvertently lead to obesity that often persists into adulthood. Studies during the pandemic have already evidenced increased adiposity in children.^12^

Physical activity is crucial for development in children. It influences their physical and mental health and their social dynamics. The 2020 WHO guidelines call for children to accumulate at least an average of 60 min of moderate-to-vigorous physical activity (MVPA) daily. They also recommend that vigorous physical activities and muscle- and bone-strengthening activities should be incorporated at least three days a week. The WHO 2020 guidelines further advocate for children to reduce the amount of recreational ST. However, there is a lack of universal policies guiding definitive ST for children.^13^ Some national guidelines recommend limiting sedentary recreational ST to no more than 2 h per day and recommend breaking up long periods of sitting as often as possible. Furthermore, a family-media use plan has been recommended to help families limit ST.^13^

Sedentary behaviour interventions require attention from both public health advocates and large community-based associations. Schools are an ideal setting to implement sedentary behaviour interventions, as children spend a great deal of time at school. Environmental adaptations can be implemented, such as standing rather than sitting at desks when using screens and devices. Leisure activities can also be introduced in the home and community. Motivational approaches can be facilitated by healthcare and social workers. Interventions should include all stakeholders who engage with this serious health concern, including parents and educators.^10,14^

## Screen time and mental well-being of children

Developments in artificial intelligence (AI), machine learning, automation and robotics will undoubtedly accelerate during this current fourth industrial revolution. The developing brains and bodies of the children and adolescents who are learning, working, ‘playing’ and socialising in this digital era will inevitably be affected simultaneously. Research on the impact of increased ST on the brains and mental well-being of children and adolescents is in its infancy. Moreover, research is confounded by many factors: age; gender; type of screen (cell phone, videogames, television, computer); duration of exposure; purpose of use (education, recreation, social); whether the activity is solitary or in the company of others, such as parents; the content of the exposure (violence, educational); active vs passive ST and socio-economic factors. Furthermore, primary and secondary effects are not always separable: some of the effects on the brain and mental (psychological, cognitive, social) health may be because of associated negative behaviours, such as poor dietary habits and increased sedentary behaviour, or the loss of other positive behaviours such as ‘green time’. A lack of physical activity, poor sleep hygiene and reduced social interaction are often closely associated with increased ST.^15,16,17,18,19^

Various types of Internet usage could possibly have different effects on the developing brain in both adverse and salutary ways, as many cognitive processes are strongly influenced by environmental factors.^20^ Currently, there is moderately strong evidence of an association between ST and symptoms of depression, but little or no evidence of an association between ST and eating disorders or suicidal ideation.^21^ An association between the length of sedentary ST and indicators of mental health, including hyperactivity/inattention problems, internalising problems, poorer psychological well-being and a poorer perceived quality of life, has been found in children and adolescents.^22^ Inattention difficulties were evident in pre-schoolers and were associated with increased ST.^4^ Screen time in children and adolescents was found to be associated with the risk of depression in a non-linear dose–response manner^23^ and to negatively impact self-esteem. It increases both the incidence and severity of mental health concerns and addictions; impedes learning and increases the risk of premature cognitive decline.^24^

While most studies to date have been cross-sectional in nature, the results of a review of 35 longitudinal studies suggested that the impact of ST on the prevalence of mental health problems amongst young people is likely to be negligible or small. The authors, however, acknowledged the need for further longitudinal studies to evaluate the relationship between ST and the internalising of mental symptoms.^25^ Similar calls for longitudinal research were made to understand the moderating role of sleep, especially in children, on the effects of ST.^26^ An editorial published by the Association for Child and Mental Health concluded that not all forms of ST have equal effects as some were not associated with adverse effects.^27^ The need for high-quality, longitudinal research that disaggregates the differential effects on different groups of users, to inform how the positive effects of ST can be harnessed, was highlighted. Importantly, the editorial noted that immediate action was necessary to protect users and to promote well-being in children.^27^

While more research is awaited, young brains and minds are developing and the potential long-term sequelae have to be considered now. It is therefore prudent for both clinicians and parents to note the guidelines compiled by the Canadian Paediatric Society^28^ to mitigate risk and promote psycho-physiologic resilience.

## Conclusion

Smartphone users need to be smart about their well-being. While embracing the benefits of a highly digitalised world, we need to simultaneously mitigate the potential risks they pose to the health of growing children. This can start with straightforward, practical behavioural lifestyle modifications. Children should spend on average 2 h a day outdoors, including at least an hour of physical activity, on at least three days a week. Annual visual assessments are necessary to rule out refractive errors, as today’s child is born into a world that has become ‘closer’ in the digital age. Some noteworthy suggestions of the Canadian Paediatric Society^28^ are as follows: start introducing age-appropriate time-based interactions with screens – no ST for infants and toddlers and children under 5 years should not have more than 1 h of ST per day; consider screen-free mealtimes; keep the television in the family room and outside the bedroom. Parents should start policing device use amongst their school-going children and should model healthy screen use themselves. They should also avoid the re-purposing of devices as pacifiers for infants and toddlers.
